# Glycosaminoglycans and Contrast Agents: The Role of Hyaluronic Acid as MRI Contrast Enhancer

**DOI:** 10.3390/biom10121612

**Published:** 2020-11-28

**Authors:** Alfonso Maria Ponsiglione, Maria Russo, Enza Torino

**Affiliations:** 1 Department of Electrical Engineering and Information Technology (DIETI), University of Naples “Federico II”, Via Claudio 21, 80125 Naples, Italy; alfonsomaria.ponsiglione@unina.it; 2 Department of Chemical, Materials and Production Engineering, University of Naples Federico II, Piazzale V. Tecchio 80, 80125 Naples, Italy; maria.russo@espci.psl.eu; 3 Interdisciplinary Research Center on Biomaterials, CRIB, Piazzale V. Tecchio 80, 80125 Naples, Italy

**Keywords:** hyaluronic acid, glycosaminoglycan, hydrogel, MRI, hydrodenticity, precision medicine

## Abstract

A comprehensive understanding of the behaviour of Glycosaminoglycans (GAGs) combined with imaging or therapeutic agents can be a key factor for the rational design of drug delivery and diagnostic systems. In this work, physical and thermodynamic phenomena arising from the complex interplay between GAGs and contrast agents for Magnetic Resonance Imaging (MRI) have been explored. Being an excellent candidate for drug delivery and diagnostic systems, Hyaluronic acid (HA) (0.1 to 0.7%*w*/*v*) has been chosen as a GAG model, and Gd-DTPA (0.01 to 0.2 mM) as a relevant MRI contrast agent. HA samples crosslinked with divinyl sulfone (DVS) have also been investigated. Water Diffusion and Isothermal Titration Calorimetry studies demonstrated that the interaction between HA and Gd-DTPA can form hydrogen bonds and coordinate water molecules, which plays a leading role in determining both the polymer conformation and the relaxometric properties of the contrast agent. This interaction can be modulated by changing the GAG/contrast agent molar ratio and by acting on the organization of the polymer network. The fine control over the combination of GAGs and imaging agents could represent an enormous advantage in formulating novel multifunctional diagnostic probes paving the way for precision nanomedicine tools.

## 1. Introduction

Glycosaminoglycans (GAGs) have always attracted the interest of many research groups because of their versatile properties, making them desirable resources for the design of multifunctional materials in biomedicine [1,2,3]. Compared to other classes of materials, like amino acid sequences, which have been coded and possess well-known properties and characteristics, GAGs represent a still unexplored group of materials, not specifically ascribable to any of the already known chemical and biophysical patterns [1]. This stimulates the investigation of their nature and behavior in biological environments, e.g., nanoscale interactions with proteins, lipids, and other GAGs, in order to fully understand and control their potential in the precision nanomedicine field as drug delivery systems and image contrast enhancers. As naturally derived biomaterials from affordable sources, GAGs represent an abundant, biodegradable, biocompatible class of materials for the synthesis of the new generation of nanomedicines, overcoming some of the toxicity- and stability-related issues of synthetic materials [4]. Physico-chemical properties of GAGs, such as monomer length, reactive groups, molecular weight, and charge, proved to be key features to design engineered nanostructures for biomedical applications [1,4]. In addition, they provide a large polymeric backbone for chemical modification where small molecules, drugs, proteins, or diagnostic agents can be easily conjugated onto the NPs’ surface or, alternatively, physically encapsulated into the NPs’ core or shell [5,6], thereby improving their targeting efficiency. The easy decoration of NPs, for example with polyethylene glycol (PEG), can prolong their in-vivo circulation time, which increases the possibility of accumulation of the delivered drug in the site of interest [7,8,9]. Additional advantages, compared to other metallic or silica NPs [10,11,12], lie in their high tunability thanks to the recent advancements in nanotechnology and material processing techniques, from batch synthesis to high-pressure homogenization and microfluidics. The latter, in particular, proved to be a scalable, low-cost, and high-throughput technique for controlling sizes, shapes, porosity, structure, and functional properties of polymer NPs [13,14,15,16,17,18,19,20]. Among the GAGs, hyaluronic acid (HA) (alone or coupled with other GAGs) proved to be an ideal candidate for designing nanostructured probes for drug delivery and imaging [4]. HA, also called hyaluronan, is an anionic highly hydrophilic GAG ubiquitously presents in tissues and fluids and composed of a repeating disaccharide of d-glucuronic acid and N-acetyl-d-glucosamine. It is present in the extracellular matrix and plays key roles in modulating cellular functions [4]. Moreover, HA can intrinsically target CD44 receptors, which are overexpressed in various tumor cells, thus serving as a targeting moiety for cancer therapy [21,22,23]. Very recent works demonstrated its potential as hydrogel nanosystem for neural tissue regeneration [5], theranostic agent in breast cancer and atherosclerosis [24,25,26], engineered nanostructure for multimodal imaging of B-cell lymphoma [27,28], and contrast enhancer in Magnetic Resonance Imaging (MRI) [29,30]. Concerning the use of HA as a contrast enhancer, studies [31,32] highlighted that MRI signal depends on the GAGs’ concentration in human tissues, especially in articular cartilage, whose synovial fluid is made up of 98% HA [33] (ranging from 0.25 to 0.4%*w/v* in healthy adults [34,35]). They showed that the administration of a paramagnetic contrast agent, a metal chelate like Gadolinium diethylene triamine pentaacetic acid (Gd-DTPA), can be used to visualize relative GAG distribution in-vivo since the negative charge of the contrast agent will distribute itself within articular cartilage in a spatially inverse relationship to the concentration of the negatively charged GAG molecules [31,32]. In a more recent study [36], crosslinked HA-based hydrogels at different HA concentrations (ranging from 17%*w*/*v* to 30%*w*/*v*) have been used as model tissues to investigate the relaxation enhancement of an MRI contrast agent interacting the hydrogel structure at increasing magnetic fields. Such studies are focused on the characteristic correlation times of the metal chelate within the hydrogel but do not take into account the thermodynamic phenomena underlying the HA-contrast agent interaction, which are crucial to understand the mixing process and control the complexation of the two compounds. Furthermore, no tissue models at low HA concentrations (below 1%*w*/*v*), which correspond to the physiological range of HA concentrations in human tissues [37,38,39], have been yet developed nor adopted. The investigation of the relaxation enhancement mechanisms in the presence of a biopolymer network can be fundamental for the rational design of novel nanostructured MRI contrast agents with enhanced properties [40] in the field of drug delivery and precision medicine [2]. Recently, in our previous work, HA-based nanostructures have been investigated [41] and the impact of the structural properties of the hydrogel matrix on the relaxometric properties of an MRI contrast agent has been explained introducing the concept of hydrodenticity, i.e., the complex equilibrium established by the elastic stretches of polymer chains, water osmotic pressure, and hydration degree of the contrast agent, able to boost the relaxometric properties of the contrast agent itself. In other previous works [42,43], we demonstrated how the HA hydrogel structural parameters can impact the relaxivity of MRI contrast agents and then we translated the acquired know-how into a microfluidic flow focusing approach to design and produce functional Gd-loaded nanohydrogels with tunable relaxivity for MRI and multimodal imaging applications [41,44,45,46]. 

Herein, based on our previous findings, we investigate from both a physical and thermodynamic perspective the interactions between HA, chosen as a GAG model, and Gd-DTPA, as a linear ionic MRI Gd-based contrast agent, able to boost the relaxometric properties of the metal-chelate. We highlight the importance of understanding and controlling their complex interplay and show how to take advantage of their combination to develop nanosystems with precisely tailored composition. In the foreseeable future, this knowledge can contribute to the innovation of traditional drugs and imaging agents.

## 2. Materials and Methods

### 2.1. Materials

Divinyl sulfone (DVS, 118.15 Da), Diethylenetriaminepentaacetic acid gadolinium (III) dihydrogen salt hydrate (Gd-DTPA, 547.57 Da) and Sodium hydroxide pellets (NaOH) are purchased from Sigma Aldrich (Milan, Italy). Sodium Hyaluronate, with an average molecular weight of 42 kDa is supplied Bohus Biotech (Strömstad, Sweden) as dry powder and used without purification. Milli-Q water is systematically used for sample preparation, purification, and analysis.

### 2.2. Sample Preparation

Non-crosslinked HA samples, from 0.1 to 0.7%*w*/*v*, are prepared by dispersing polymer powder in Milli-Q water and then mechanically mixed using a magnetic stirrer (Fisher Scientific Italia, Milan, Italy), 500 rpm at Room Temperature (RT) for 2 h. Crosslinked HA samples are prepared by adding 0.2 M NaOH to the above-described solutions in order to achieve the desired pH for the crosslinking reaction and samples are mechanically stirred for 2 h (RT, 500 rpm). DVS is then added, with a DVS/HA weight ratio ranging from 2 to 11, to chemically crosslink the polymer network. The crosslinking reaction is performed at RT for 24 h in order to obtain a homogeneous gel. The biocompatibility of HA–DVS hydrogels is already confirmed in the literature [47]. Crosslinked and non-crosslinked Gd-DTPA loaded samples are prepared by adding Gd-DTPA at a concentration ranging from 0.01 and 0.2 mM (0.01—0.02—0.03—0.04—0.05—0.06—0.08—0.1—0.13—0.15—0.18—0.2 mM).

### 2.3. Time-Domain Relaxometry at 20 MHz and 60 MHz

Bruker Minispec (Bruker, Billerica, MA, USA) mq20 and mq60 bench-top relaxometer operating at 20 MHz (magnetic field strength: 0.47 T) and 60 MHz (magnetic field strength: 1.41 T), respectively, are used to measure longitudinal relaxation times (T1). 1 mL and 300 µL of the prepared samples are used for the measurements at 20 and 60 MHz, respectively. Samples are placed into the NMR probe for about 15 min for thermal equilibration. T1 values are determined by both saturation (SR) and inversion recovery (IR) pulse sequences. The relaxation recovery curves are fitted using a multi-exponential model. Relaxivity, r1, is calculated from the slope of the regression line of the relaxation rate, R1 = 1/T1, versus HA concentration with a least-squares method, as showed in the following Equation (1):

R1_HA_ = R1_water_ + r1*[HA], (1)

where R1_HA_ is the relaxation rate of the HA sample expressed in s^−1^, R1_water_ is the relaxation rate of free water expressed in s^−1^, and [HA] is the polymer concentration expressed in %*w*/*v*.

### 2.4. Measurement of Water Self-Diffusion Coefficient at 20 MHz

Diffusion measurements of water molecules are carried out on a Bruker Minispec (Bruker, Billerica, MA, USA) mq 20 bench-top relaxometer using a pulsed-field gradient spin echo (PFG-SE) sequence [48]. As previously described [42], the water self-diffusion coefficient, D, is calculated by linear regression of the echo attenuation versus the tunable parameter of the PFG-SE sequence, k, as showed in the following Equation (2):

k = (γgδ)*(Δ − δ/3)(2)

where γ is the proton’s gyromagnetic ratio (equal to 42.58 MHz T^−1^), δ is the length of the two gradients (set equal to 0.5 ms), g is the strength of the two gradients (varied between 0.5 and 2 T m^−1^), Δ is the delay between the two gradients (set equal to 7.5 ms).

### 2.5. Diffusion-Ordered NMR Spectroscopy (DOSY) at 600 MHz

As described in our previous work [44], Diffusion-ordered NMR Spectroscopy (DOSY) measurements are carried out on a Varian Agilent NMR spectrometer (Agilent Technologies, Santa Clara, CA, USA) operating at 600 MHz. Gradient strengths (Gz) are varied from 500 to 32,500 G/cm. The gradient pulse duration (δ) is kept constant to 2 ms while the diffusion delay (Δ) is increased from 7 to 1000 ms. After Fourier transformation and baseline correction, DOSY spectra are processed and analysed using Varian software VNMRJ (Agilent Technologies, Santa Clara, CA, USA) in order to obtain the values of water self-diffusion coefficient, which is then plotted as a function of Δ.

### 2.6. Isothermal Titration Calorimetry

Titration experiments are performed by using a Nano ITC Low Volume calorimeter from TA Instruments (New Castle, DE, USA) in accordance with our previously adopted protocol [44]. The sample cell (700 µL) and the syringe (50 µL) are filled with aqueous solutions of HA (from 0.1 to 0.7%*w*/*v*) and Gd-DTPA (1.5 mM) respectively. Injection volumes and intervals are fixed at 1 µL and 500 s, respectively. Measurements are performed at 25 °C with a stirring rate of 200 rpm. Analysis and modeling of the raw data is carried out using the NanoAnalyze (TA instruments, New Castle, DE, USA). The function adopted to analyze the ITC data is the sum of two models: independent sites model plus a constant used for the blank (i.e., Gd-DTPA in water). The first point is excluded from the analysis. Statistics of the thermodynamic parameters are calculated on 1000 trials with a confidence level equal to 95%.

## 3. Results and Discussion

Longitudinal relaxation times, T1, measured both at 20 MHz and 60 MHz of HA solutions at increasing DVS/HA mass ratio, is reported in Table 1.

The experiments show a measurable decrease in T1 at increasing DVS/HA mass ratio, which is more evident at low frequency (20 MHz) when T1 is lower. Indeed, in the range of magnetic field between 0.3 T and 3 T, which is the range of preclinical and clinical MRI applications, T1 increases with the field strength. Therefore, if we read Table 1 horizontally, we will notice the appreciable increase in T1 due to the increase in the magnetic field. This phenomenon occurs because the Larmor frequency scales with field strength and, with increasing Larmor frequencies, the fraction of protons able to interact at the higher Larmor frequency decreases, resulting in longer T1 values.

**Figure 1:**
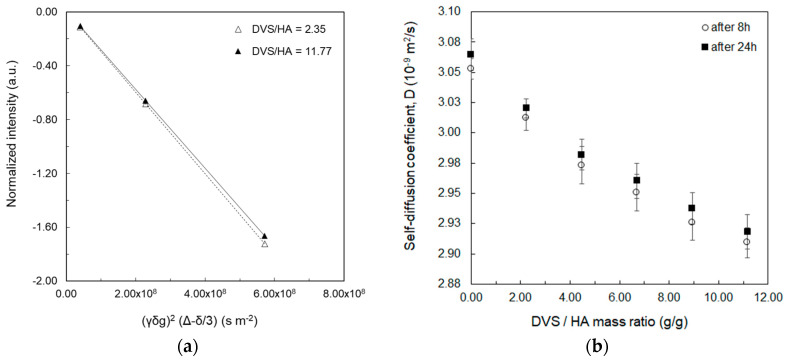
Diffusion measurements at 20 MHz: (**a**) Stejskal-Tanner plot to calculate water self-diffusion coefficient of 0.25%*w*/*v* HA crosslinked with DVS at 2.35 (empty triangles) and 11.77 (filled triangles) DVS/HA mass ratio after 8 h from the addition of the crosslinker; (**b**) Self-diffusion coefficient as a function of DVS/HA mass ratio measured after 8 h (empty circles) and 24 h (filled squares) from the addition of the crosslinker.

Then, water self-diffusion coefficient was measured through a Stejskal-Tanner plot (Figure 1a) for each sample reported in Table 1. Diffusion values, *D*, are also plotted against the DVS/HA mass ratio (HA fixed at 0.25%*w*/*v*) in Figure 1b, where the diffusion is measured 8 h and 24 h after the addition of the crosslinker (see also Table S1 of the Supplementary Material).

In Figure 1a, water self-diffusion coefficient is determined by the slope of the straight line as already reported elsewhere [49,50,51]. A reduction in the water mobility with increasing DVS/HA mass ratio can be observed in Figure 1a by looking at the increasing slope of the two regression lines of the Stejskal-Tanner plot. This is far more evident in Figure 1b where an inverse relationship can be observed between the water self-diffusion coefficient and DVS/HA mass ratio, as it also results from studies of solvent molecules within polymer matrices or in confined environments [11,52]. A time-dependent effect of the crosslinking reaction on the mobility of water molecules can be also observed in Figure 1b by comparing *D* values at 8 h and 24 h. Indeed, at 8 h, the crosslinking reaction is completed [53,54] and the swelling process is ongoing with polymer chains slowly hydrating and relaxing, thus the rate of water diffusion in the polymer networks is still slow while the hydrogel matrix is hydrating [55], binding, and entrapping water molecules. On the other hand, at 24 h, the swelling process is in the later stage, all the sulfonyl-bis-ethyl bridges between the hydroxyl groups of the HA are formed, polymer chains are well-relaxed and the swelling equilibrium is almost reached, as observed in previous studies on swelling time of crosslinked HA [55], i.e., a balance between bound water and bulk water is achieved, thus the contribution from the free diffusing water molecules is higher and the average self-diffusion coefficient assumes slightly higher values than those measured after 8 h [49]. However, this difference is not significantly appreciable especially with growing DVS/HA, since the higher crosslinking density limits polymer chains movement, thus lowering the water uptake and shortening the time to reach the swelling equilibrium [56,57].

**Figure 2:**
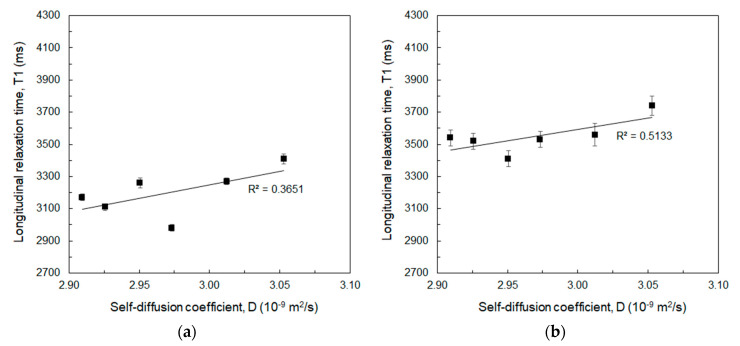
Longitudinal relaxation time (T1) as a function of the water self-diffusion coefficient measured for DVS/HA mass ratio solutions without Gd-DTPA at: (**a**) 20 MHz; (**b**) 60 MHz. Linear regression lines with values of the determination coefficients are displayed.

T1 changes with water self-diffusion coefficient are evaluated afterwards at 20 MHz and 60 MHz, as shown in Figure 2a,b respectively.

Both Figure 2a,b show how T1 increases with increasing water self-diffusion coefficient. This is due to the higher mobility of the water slowing down the time taken by protons to re-align to the external magnetic field after the stimulation with controlled radiofrequency pulses of the SR sequence. Higher T1 values in Figure 2b compared to Figure 1a are due to the increase of T1 with the applied magnetic field, as from previous considerations about Table 1. It is worth noting how Figure 1 and Figure 2 show the opportunity to obtain a relaxation enhancement by simply increasing the crosslinking degree of the sample, which is responsible for the reduction in the water mobility that thereby shortens the T1.

**Figure 3:**
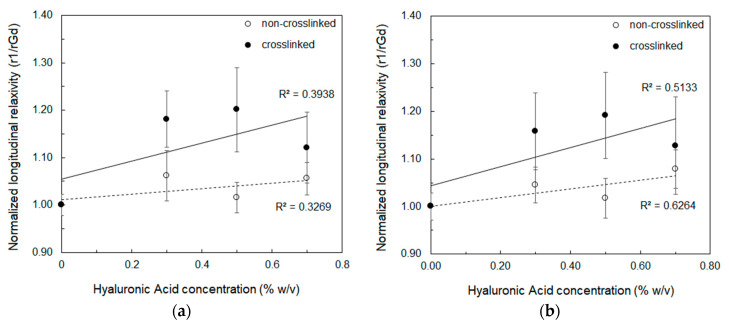
Longitudinal relaxivity (r1) for crosslinked (filled circles) and non-crosslinked (empty circles) samples with addition of Gd-DTPA, normalized against the relaxivity of free Gd-DTPA (rGd), as a function of the HA concentration measured at 60 MHz using: (**a**) Saturation Recovery sequence; (**b**) Inversion Recovery sequence. Linear regression lines with values of the determination coefficients are displayed.

The further step of our experimental campaign consisted in measuring the relaxivity, r1, as defined from Equation (1), for crosslinked and non-crosslinked samples with addition of Gd-DTPA. Here, DVS/HA is kept equal to 8 and three different HA concentrations are tested: 0.3%*w*/*v*, 0.5%*w*/*v*, and 0.7%*w*/*v*. Results of measurements carried out with SR and IR sequences are plotted in Figure 3a,b respectively (see also Table S1 of the Supplementary Material for the measured longitudinal relaxation times). The r1 values of the samples are normalized against the longitudinal relaxivity of free Gd-DTPA in water (rGd). As a reference, relaxivity of crosslinked and non-crosslinked samples without Gd-DTPA are reported in the Table S3 of the Supplementary Material.

Both SR and IR sequences confirm that r1 increases with the polymer concentration and the values are slightly higher (up to 1.2 folds) than the relaxivity of free Gd-DTPA in water. The higher accuracy of the IR sequence explains the higher determination coefficient (R^2^) of the linear regression lines displayed in the both graphs. These values are two and three orders of magnitude higher than the relaxivity of the polymer without Gd-DTPA (see Tables S2 and S3 of the Supplementary Material).

This behavior is explained by the reorientation and residence times of the water molecules interacting with HA. At increasing HA concentration, indeed, the collisions of water molecules and neighboring polymer chains increase the microviscosities of the environment [58] and the percentage of the water molecules with the longest correlation time increases with respect to those with shortest, with a consequent boost in the relaxivity [36]. Hence, the water molecules reorient more slowly, and a stronger influence of the bound water molecules on the water relaxation is expected [58].

**Figure 4:**
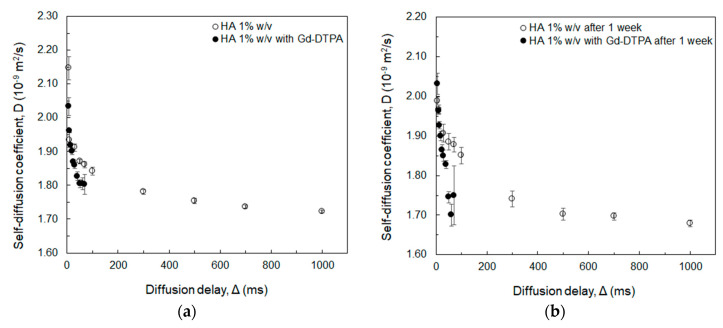
NMR-DOSY measurements of water self-diffusion coefficient at 600 MHz as a function of the diffusion delay in: (**a**) 1%*w*/*v* HA with and without Gd-DTPA at 18 mM; (**b**) measurements repeated after one week.

Moreover, the presence of Gd-DTPA has a significant impact on the relaxivity by further decreasing the water self-diffusion coefficient within the polymer network. This influence of Gd-DTPA is studied at increasing diffusion delays and observation times through NMR DOSY experiments, as displayed in Figure 4.

Both Figure 4a,b show that the co-existence of polymer and Gd-DTPA affects the water mobility more than the polymer alone, causing an additional reduction in the value of the water self-diffusion coefficient. Compared to our previous study [44], where the mobility of water molecules in presence of HA and Gd-DTPA was investigated at low contrast agent concentrations (below 30 µM), here a relatively high Gd-DTPA concentration (18 mM) is used in order to amplify the impact of the contrast agent on the water self-diffusion coefficient. However, due to the interference of Gadolinium with NMR measurements [59,60], the highest diffusion delay in the case of Gd-DTPA samples was 70 ms, since values above this threshold present a very low signal-to-noise ratio impairing the reliability of the taken measurements. Therefore, up to Δ = 70 ms, which is enough to describe the movement of water molecules within the polymer meshes in the micrometer range (a Δ range from 1 ms to 70 ms corresponds to diffusion distances from 0.5 µm to 40 µm) [61,62,63,64,65,66], the Gd-DTPA causes a further decrease in the solvent mobility. This is also confirmed after one week (Figure 4b), where a more evident drop in the water self-diffusion occurs in presence of Gd-DTPA at longer diffusion delay (50 ms < Δ < 70 ms), i.e., nearby the polymer chains.

**Figure 5:**
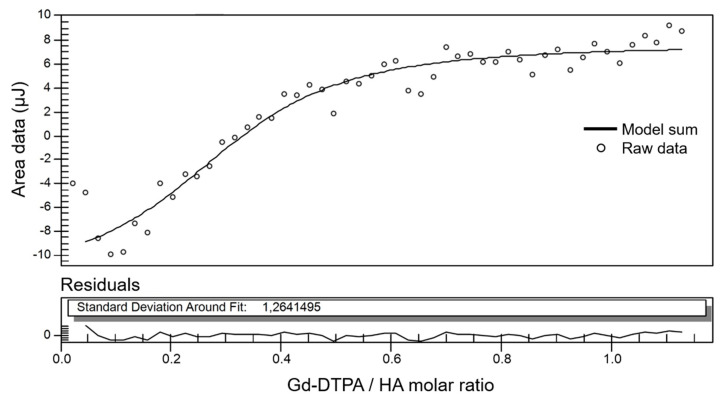
Fitting of ITC data for Gd-DTPA titrated into 0.4%*w*/*v* HA. ITC peak area data (empty circles) are plotted as a function of the Gd-DTPA/HA molar ratio. The model curve (solid line) is calculated as the sum of two models: independent sites model plus a constant used for the blank (i.e., Gd-DTPA in water). Residuals of the model and the standard deviation around fit are displayed in the bottom graph.

The impact of Gd-DTPA was carried out also from a thermodynamic perspective by investigating the mixing between Gd-DTPA and HA through ITC. The modeling of the collected ITC data is showed in Figure 5 and Table 2.

A representative binding isotherm for the titration of the HA with Gd-DTPA is shown for a single HA concentration (equal to 0.4%*w*/*v*). The binding curve shows a slow increase in the enthalpy of binding for the injections before 0.5 mol of Gd-DTPA per mole of HA. After this, a change in the signal is observed for the following injections with the curve reaching a constant value after saturation of the binding sites in the polymer chain, similarly to what is showed in previous works on the synthesis of metal-chelating polymers [67,68]. Similar ITC curves are obtained for the other tested concentrations (from 0.2 to 0.7%*w*/*v*) with the exception of HA = 0.1%*w*/*v*, which is showed in the Supplementary Material (Figure S1).

Since multiple binding sites are usually present on GAGs [69], thermodynamic parameters are determined by using an independent sites model, i.e., assuming multiple independent sites, and a constant used to model the blank (Gd-DTPA in water). The best fit of the ITC curve gives the following parameters: constant for the blank; reaction stoichiometry or number of binding sites (n); dissociation constant (Kd); enthalpy gain (ΔH); entropy gain (ΔS).

Table 2 includes the thermodynamic parameters (mean and standard deviation) calculated at increasing HA concentrations.

For HA concentrations above 0.1%*w*/*v*, the interaction process is exothermic (ΔH < 0); the binding affinity between the HA and Gd-DTPA, expressed by 1/K_d_, is relatively weak and ranges from 0.1 × 10^6^ to 4 × 10^6^ M^−1^; the reaction stoichiometry n ranges from 0.2 to 0.7 and decreases with the polymer concentration. The fitting parameters allows the calculation of the Gibbs free energy (ΔG = ΔH − T∙ΔS, being T the temperature), showing the spontaneous nature of the interaction (ΔG > 0), which is mainly driven by the entropy (|ΔH| < |T∙ΔS|).

The favorable enthalpy conditions (ΔH < 0) suggest the formation of new complexes also encouraged by the conformational changes of the HA in presence of Gd-DTPA, which increases the entropy of the ternary system (ΔS > 0). Despite the interaction process being both entropically and enthalpically favorable, nor the enthalpy nor the entropy gains are significantly influenced by the increase in polymer concentration. Conversely, the reaction stoichiometry shows an inverse relationship with the investigated HA concentrations. This can be attributed to the entanglement and conformational changes of the polymer, which are highly dependent on concentration. Indeed, at HA concentrations higher than 0.1%*w*/*v*, HA chains form a continuous three-dimensional network [37,39] with chains interacting with each other and forming stretches of double helices that makes the network more rigid and increases the fraction of water bound to HA chains and confined within the polymer matrix with respect to the free water not interacting with the polymer [70]. Therefore, the higher the polymer concentration is, the more HA-HA entangles and HA-water hydrogen bonds are formed, thus reducing the number of available sites, n, for the interaction with Gd-DTPA. As already observed in charged hydrophilic GAG [71], the interplay between intra- and inter-molecular solvent hydrogen bonding, along with the entanglement mechanism, plays a major role during interaction processes, and is also responsible for new arrangements of the polymer chains in solution [72].

However, the hydrogen bond network developed in solution around a polysaccharide depends not only on the water layers organization but also on the presence of other solute species capable of hydrogen bonds formation [72], like Gd-DTPA in this study. It is also known that a drug binding to a GAG is expected to cause a decrease in the internal degrees of freedom of the GAG, thus affecting its possible conformational changes [73]. Therefore, in such a continuous reorganization of the HA chains in water, the presence of Gd-DTPA provides an additional contribution confirmed by the large positive entropy changes, which arises from the conformational freedoms of both HA and Gd-DTPA upon mixing, as also measured in other studies on Gd complexation [74].

The entropic gain due to conformational changes predominates but is not the only phenomenon governing the process, since a smaller but still significant enthalpic contribution is measured and suggests the presence of weak interactions between HA and Gd-DTPA, ascribable to non-covalent binding, namely hydrogen bond, hydrophobic, electrostatic, and van der Waals interactions [74,75]. In accordance with Flory’s mean field theory [75], such non-covalent interactions are crucial in determining the swelling equilibrium of the polymer network. Therefore, changes in the Gibbs free energy of the system can be interpreted as function of the polymer–solvent mixing, the elastic deformation of the polymer matrix, and the osmotic pressure due to the gradients of solute concentrations.

Among these interactions, the nature of the compounds in solution, both hydrophilic and negatively charged, brings our attention mainly to hydrogen bonding and electrostatic forces.

As also demonstrated elsewhere [76], hydrogen bonding phenomena is a fundamental factor determining the thermodynamics of polymers in aqueous solution and, as previously mentioned, the high hydrophilicity of HA enables the formation of inter- and intra-molecular hydrogen bonds [77]. At the beginning of the titration (Figure 5), the exchanged heat increases since the fraction of HA−water hydrogen bonds increases. Starting from a Gd-DTPA/HA ratio of 0.5, the heat decreases because other interactions take place (intra-molecular HA hydrogen bonding and HA conformational changes) giving opposite enthalpy contributions and bringing the curve to its plateau, when all HA binding sites are saturated and only water−water hydrogen bonding occur. This behavior agrees with observation reported in other studies [78], demonstrating that the enthalpy of mixing of polyelectrolyte complexes decreases at increasing salt concentration and polyelectrolyte complexation is essentially entropy driven. Furthermore, the capability of forming intra-molecular hydrogen bonds impact on the exchange and diffusion of water molecules in the inner and outer coordination sphere of the Gd-DTPA, both factors being responsible for the relaxation enhancement of the metal chelate [36,79].

As far as the electrostatic forces, it is known that the hundreds of negative charges fixed to each polymer chain are responsible for electrostatic interactions with surrounding molecules [39]. These electrostatic interactions play an important role in the mixing process, giving a large positive contribution to the entropy of the system [37,73]. In our system, we can hypothesize that intra-molecular interactions and conformational changes are driven by the presence of Gd-DTPA. Indeed, since HA and Gd-DTPA are both negatively charged, the addition of the contrast agent in solution causes electrostatic repulsion, forcing the HA chains to rearrange in order to reach a new energetically favorable configuration. This agrees with studies on the Gd-DTPA distribution in cartilage [32], showing how the negative fixed charge density of GAGs forces the contrast agent to accumulate more into areas with less GAG concentration. Moreover, as observed in other studies on polyelectrolytes interaction [80], at high enough solute concentrations, a repulsion effect can also be caused by hydration forces. These forces promote the local structuring of several layers of water molecules around the polymer due to electrostatic and hydrogen-bonding interactions. When Gd-DTPA approaches closely to the polymer, a collective disruption of these structured water layers would cost a fair amount of energy, thus producing repulsive forces.

A further non-negligible effect that needs to be taken into account is the osmotic pressure. As it occurs for solutes moving inside and outside the polymer network [75], the presence of Gd-DTPA not only induces rearrangement of the polymer in solution but also generates an osmotic pressure due to clustering of HA chains. Like other GAGs [1], indeed, HA conformational changes create regions of high anionic charge leading to high osmotic pressure, which promotes the taking up of unbound water molecule from the environment and drives the swelling of the polymer matrix [75]. The water uptake is confirmed by the large entropic contributions due to the large number of possible configurations upon swelling [58]. These changes in the osmotic pressure impact the hydration of the contrast agent and contribute to the attainment of that complex equilibrium, called hydrodenticity [41], able to boost the relaxometric properties of the Gd-DTPA, whose enhancement is promoted by the formation of the Gado-mesh, as extensively defined in previous publications [41,44].

## 4. Conclusions

In this work, diffusion (Figure 1, Figure 2 and Figure 4), thermodynamic (Figure 5 and Table 2), and relaxation properties (Table 1 and Figure 3) of HA and Gd-DTPA mixtures have been presented and discussed.

Following the previous studies on the binding between drugs and GAGs [73] and similarly to what is shown about polyelectrolyte-protein interactions [81], our results suggest that the interaction between HA and Gd-DTPA is mainly mediated by the role played by the water and determined by two factors: (i) non-covalent processes (hydrogen bonding and electrostatic forces); (ii) conformational changes of the polymer. While the former is endothermic and characterized by negative enthalpy gain, the latter is exothermic and brings a positive entropy gain. Since they occur simultaneously, the overall interaction can be described as the combination of the two above-mentioned factors with one predominating on the others during the mixing. Indeed, both HA and Gd-DTPA have the capacity of forming hydrogen bonds and coordinate water molecules, which not only produces conformational changes but also affect the relaxometric properties of the contrast agent.

In conclusion, our results show a representative picture on GAGs interaction with MRI contrast agents and contribute to build a useful framework for the interpretation of their behavior in solution and for the understanding of the fundamental phenomena underlying the MRI relaxation enhancement. Moreover, we also expect that our results can be extended to other liner Gd-based contrast agents since they present analogous chemistry and relaxation mechanisms. Further potential applications extended also to macrocyclic Gd-based contrast agents could be explored in future works. This knowledge could provide insights into the fields of nanomedicine and precision medicine, where the proper choice and combination of GAGs with imaging or therapeutic agents is the key factor for the formulation of effective targeted drug delivery systems.

## Figures and Tables

**Table 1:** Longitudinal relaxation time (T1) at different DVS/HA mass ratio.

HA (%*w*/*v*)	DVS/HA (g/g)	T1 (ms) ^1^ Mean ± std	T1 (ms) ^2^ Mean ± std
0.25	0	3410 ± 30	3740 ± 60
0.25	2.35	3270 ± 20	3560 ± 70
0.25	4.70	2980 ± 20	3530 ± 50
0.25	7.06	3260 ± 30	3410 ± 50
0.25	9.42	3110 ± 20	3520 ± 50
0.25	11.77	3170 ± 20	3540 ± 50

^1^ measured at 20 MHz. ^2^ measured at 60 MHz.

**Table 2:** Thermodynamic parameters from the modeling of ITC measurements conducted at 25 °C.

HA (%*w*/*v*)	Blank ^1^ Mean ± std ^2^	n ^1^ Mean ± std ^2^	K_d_ (*10^−6^ M) ^1^ Mean ± std ^2^	ΔH (kJ/mol) ^1^ Mean ± std ^2^	T∙ΔS (kJ/mol) ^1^	ΔG (kJ/mol) ^3^
0.1	−25.43 ± 24.36	9.99 ± 3.77	26.30 ± 722.3	24.04 ± 49.60	50.15	-26.11
0.2	5.59 ± 4.02	0.71 ± 0.37	7.25 ± 0.58	−10.12 ± 53.83	19.21	29.33
0.3	7.76 ± 1.21	0.30 ± 0.11	3.62 ± 0.12	−10.56 ± 20.03	20.49	31.05
0.4	7.37 ± 0.96	0.33 ± 0.038	3.71 ± 3.84	−11.54 ± 2.84	19.44	30.98
0.5	−2.18 ± 1.16	0.18 ± 0.040	1.60 ± 7.98	−10.06 ± 9.43	23.01	33.07
0.6	9.97 ± 22.97	0.39 ± 0.53	0.24 ± 0.013	−23.84 ± 64.48	2.48	26.31
0.7	1.56 ± 1.71	0.20 ± 0.031	1.96 ± 5.16	−11.79 ± 5.11	20.78	32.57

^1^ Values obtained by fitting ITC data with the independent sites model (constant is used for the blank, i.e., Gd-DTPA in water). ^2^ Statistics are calculated on 1000 trials with a confidence level equal to 95%. ^3^ ΔG = ΔH − T∙ΔS.

## Supplementary Materials

The following are available online at https://www.mdpi.com/2218-273X/10/12/1612/s1, Figure S1: Fitting of ITC data for Gd-DTPA titrated into 0.1%*w*/*v* HA, Table S1: Values of water self-diffusion coefficient at different DVS concentrations measured at 20 MHz after 8 h and 24 h from DVS addition, Table S2: Longitudinal relaxation times of the crosslinked and non-crosslinked samples without Gd-DTPA measured with Saturation and Inversion Recovery sequences, Table S3: Relaxivity of the crosslinked and non-crosslinked samples without Gd-DTPA measured with Saturation and Inversion Recovery sequences.

## Acronyms

DVS	Divinyl Sulfone
GAG	Glycosaminoglycan
HA	Hyaluronic Acid
IR	Inversion Recovery
ITC	Isothermal Titration Calorimetry
MRI	Magnetic Resonance Imaging
NMR	Nuclear Magnetic Resonance
NMR-DOSY	Diffusion-ordered NMR Spectroscopy
NPs	Nanoparticles
PFG-SE	Pulsed-Field Gradient Spin Echo
RT	Room Temperature
SR	Saturation Recovery
