# Supplementary material for: Glycosaminoglycans and Contrast Agents: The Role of Hyaluronic Acid as MRI Contrast Enhancer
Source: Biomolecules. 2020 Nov 28;10(12):1612. doi: 10.3390/biom10121612 (PMC7759866; doi:10.3390/biom10121612)
Supplement: Supplementary file 1 [file biomolecules-10-01612-s001.pdf]

SUPPLEMENTARY MATERIAL

Glycosaminoglycans and Contrast Agents: The Role of Hyaluronic Acid as MRI Contrast Enhancer

Alfonso Maria Ponsiglione [1,2], Maria Russo [2,†], and Enza Torino [2,3] *

1 Department of Electrical Engineering and Information Technology (DIETI), University of Naples "Federico II", Via Claudio 21, 80125, Naples, Italy; alfonsomaria.ponsiglione@unina.it
2 Department of Chemical, Materials and Production Engineering, University of Naples Federico II, Piazzale V. Tecchio 80, 80125 Naples, Italy; maria.russo@espci.psl.eu
3 Interdisciplinary Research Center on Biomaterials, CRIB, Piazzale V. Tecchio 80, 80125 Naples, Italy;
* Correspondence: enza.torino@unina.it; Tel.: +39-328-955-8158
† Present address: Microfluidique, MEMS et Nanostructures, Institut Pierre-Gilles de Gennes, CNRS UMR 8231, ESPCI Paris and Paris Sciences et Lettres (PSL) Research University, Paris 75005, France;

Table S1. Values of water self-diffusion coefficient at different DVS concentrations measured at 20 MHz after 8h and 24h from DVS addition.

HA (% w/v)	DVS/HA (g/g)	D (10^{-9} m^2/s) [1] mean ± std	D (10^{-9} m^2/s) [2] mean ± std
0.25	0	3.05 ± 0.01	3.06 ± 0.01
0.25	2.35	3.01 ± 0.01	3.02 ± 0.01
0.25	4.70	2.97 ± 0.02	2.98 ± 0.01
0.25	7.06	2.95 ± 0.02	2.96 ± 0.01
0.25	9.42	2.93 ± 0.01	2.94 ± 0.01
0.25	11.77	2.91 ± 0.01	2.92 ± 0.01

[1] after 8h from the addition of DVS. [2] after 24h from the addition of DVS.

Table S2. Longitudinal relaxation times of the crosslinked and non-crosslinked samples without Gd-DTPA measured with Saturation and Inversion Recovery sequences.

HA (% w/v)	HA (mM)	DVS/HA (g/g)	T1 (ms) [1] mean ± std	T1 (ms) [2] mean ± std
0	0	0	3650 ± 10	3770 ± 40
0.3	0.071	0	3730 ± 10	3790 ± 20
0.3	0.071	8	3500 ± 10	2926 ± 7
0.5	0.12	0	3630 ± 10	3660 ± 10
0.5	0.12	8	3000 ± 10	3018 ± 7
0.7	0.17	0	3640 ± 10	3770 ± 40
0.7	0.17	8	3290 ± 20	3321 ± 7

[1] measured using a Saturation Recovery (SR) sequence. [2] measured using an Inversion Recovery (IR) sequence.

Table S3. Relaxivity of the crosslinked and non-crosslinked samples without Gd-DTPA measured with Saturation and Inversion Recovery sequences.

Sample	DVS/HA (g/g)	r1 ($mM^{-1}s^{-1}$) [2] mean ± std	r1 ($mM^{-1}s^{-1}$) [2] mean ± std
non-crosslinked	0	0.014 ± 0.032	0.017 ± 0.041
crosslinked	8	0.26 ± 0.18	0.22 ± 0.30

[1] measured using a Saturation Recovery (SR) sequence. [2] measured using an Inversion Recovery (IR) sequence.

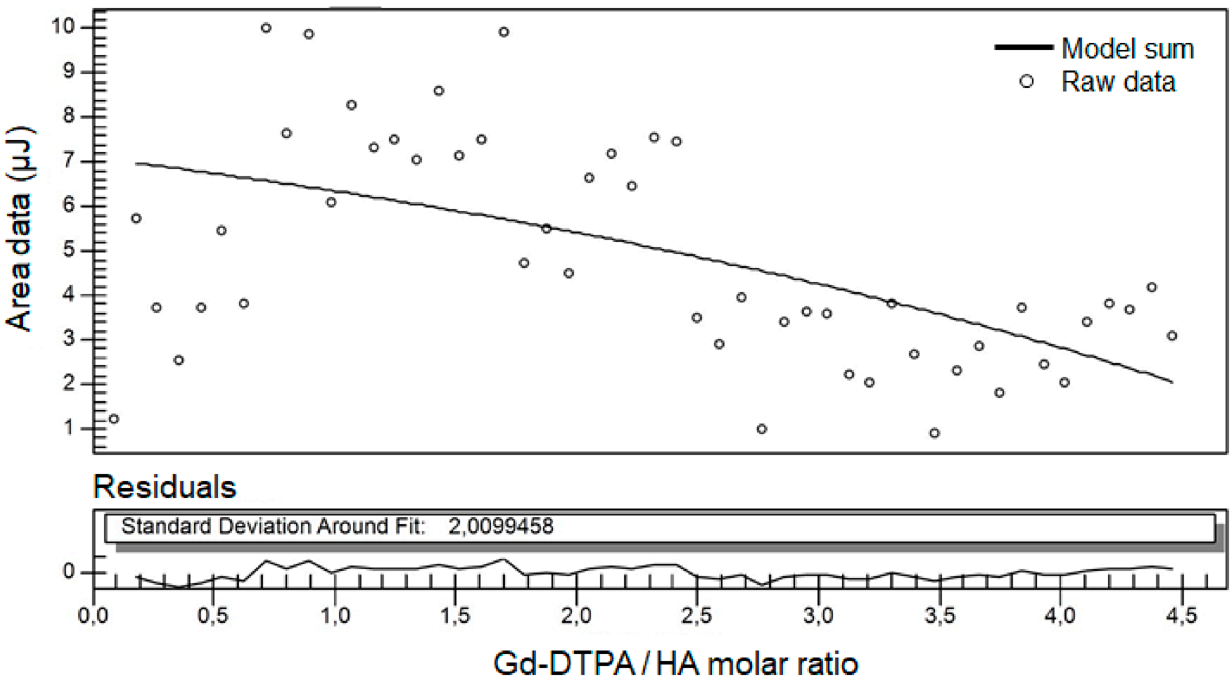

Figure S1. Fitting of ITC data for Gd-DTPA titrated into 0.1%w/v HA. ITC peak area data (empty circles) are plotted as a function of the Gd-DTPA / HA molar ratio. The model curve (solid line) is calculated as the sum of two models: independent sites model plus a constant used for the blank (i.e. Gd-DTPA in water). Residuals of the model and the standard deviation around fit are displayed in the bottom graph.
